# Adherence to Healthy Dietary Patterns and Glioma: A Matched Case-Control Study

**DOI:** 10.3390/nu15234886

**Published:** 2023-11-23

**Authors:** Weichunbai Zhang, Yongqi He, Ce Wang, Feng Chen, Bo Jiang, Wenbin Li

**Affiliations:** Department of Neuro-Oncology, Cancer Center, Beijing Tiantan Hospital, Capital Medical University, Beijing 100070, China; zwchunbai@163.com (W.Z.); 122021010536@mail.ccmu.edu.cn (Y.H.); 13146851017@163.com (C.W.); chenfeng@bjtth.org (F.C.); jiangboprof@163.com (B.J.)

**Keywords:** healthy dietary pattern, glioma, principal component analysis, dose–response relationship, matched case-control study

## Abstract

Recent studies have revealed a putative relationship between diet and glioma development and prognosis, but few studies have examined the association between overall diet and glioma risk. This study, conducted in China, employed a hospital-based case-control approach. The researchers utilized an a priori method based on dietary data to evaluate compliance scores for five healthy dietary patterns (the Mediterranean diet, the Dietary Approaches to Stop Hypertension (DASH) diet, the Mediterranean-DASH diet Intervention for Neurodegenerative Delay (MIND) diet, the Paleolithic diet, and the Planetary Health Diet) in 1012 participants. At the same time, data-driven methods were used to explore the association between dietary patterns and glioma via principal component analysis (PCA). In the multivariate model, adhering to the Mediterranean diet (odds ratio (OR) = 0.29; 95% confidence interval (95% CI): 0.17–0.52), the DASH diet (OR = 0.09; 95% CI: 0.04–0.18), the MIND diet (OR = 0.25; 95% CI: 0.14–0.44), and the Paleolithic diet (OR = 0.13; 95% CI: 0.06–0.25) was associated with a reduced glioma risk. The results of PCA suggested that increasing the intake of plant-based foods and fish and limiting foods rich in carbohydrates, fats, and salts were associated with a reduced glioma risk. There was a substantial nonlinear dose–response association between glioma and the Mediterranean diet score. However, the DASH diet score, the MIND diet score, and the Paleolithic diet score exhibited linear dose–response relationships. Therefore, this study finds that dietary patterns may be an influencing factor for glioma risk.

## 1. Introduction

The most frequent type of malignant adult brain tumors are gliomas, which account for 80% of all cases [1,2]. Even with aggressive treatment, the prognosis of glioma remains poor, with a median overall survival of fewer than 20 months and a 7% 5-year survival rate in certain glioblastoma patients [2]. The aggressive nature of gliomas makes it difficult to entirely eliminate them by surgical treatment [3]. A significant illness burden is placed on patients and families as a result of the high death rate of gliomas, rapid disease development, and ease of recurrence. As a result, glioma prevention has emerged as a crucial disease-fighting strategy. Identification of modifiable factors in primary prevention remain a typical goal in epidemiological studies of gliomas [4].

Diet has emerged as a significant risk factor for developing cancer [5]. The World Health Organization claimed that 30% of cancer cases in industrialized nations might be related to food [6]. A lack of vegetables and fruits and prolonged consumption of processed meats were thought to be risk factors for the majority of human cancers [7,8]. Therefore, gliomas are no exception [9]. Studies have shown that many food groups such as vegetables [10], cured meat [11], grains [12], coffee [13], and tea [14] are closely related to glioma. However, most studies on glioma have focused on single food groups or nutrients, while diets are much richer and more complex. When considering the association between a certain food group and disease, we can ignore the interactions and combined exposures between different foods [15]. Therefore, measuring the combination of dietary combinations by analyzing dietary patterns is more relevant than that of individual food groups [16] and also provides more direct evidence for the development of dietary guidelines [16]. In this regard, studies in recent years have shown that common diets, such as Mediterranean or Westernized diets, are related to other types of cancer, such as skin [17], lung [18], and stomach [19] cancer; however, dietary patterns and gliomas have hardly ever been studied. Only one Iranian study has demonstrated a significant risk reduction of glioma by adhering to the Mediterranean diet (odds ratio (OR) = 0.36; 95% confidence interval (95% CI): 0.16–0.78) [20]. However, a US cohort study did not find any substantial association [21].

To further explore the impact of dietary patterns on glioma, we conducted an analysis based on the Chinese population, to provide scientific support for the creation of primary glioma prevention measures.

## 2. Materials and Methods

### 2.1. Study Population

Our methodology has been extensively detailed in prior studies [22]. In brief, this case-control study was conducted in 2022 and focused on the Chinese adult population. According to the latest diagnostic criteria for glioma [23], the cases were glioma patients who visited Beijing Tiantan Hospital and were collaboratively diagnosed by pathologists and neuro-oncologists; they did not exceed several months from diagnosis to investigation. The control population consisted of healthy people of the community who were paired 1:1 based on age (±5 years) and sex. After excluding the population who refused to participate in the study (*n* = 56), the response rate was 97% in the case group and 93% in the control group. After further eliminating individuals with cognitive impairments who were unable to complete the questionnaire (*n* = 4), incomplete pathological information (*n* = 2), incomplete questionnaire information (*n* = 11), and mismatched individuals (*n* = 9), a total of 506 pairs were included in the statistical analysis (Figure S1). After a written and oral explanation of the study methods, all participants agreed and accepted a questionnaire survey to collect relevant data. The questionnaire data mainly included basic information and dietary information. Beijing Tiantan Hospital’s Institutional Review Board approved the study (No. KY2022-203-02).

The inclusion criteria mainly include the following: (a) pathological diagnosis of glioma (limited to case groups) and (b) above 18 years of age. Exclusion criteria include the following: (a) significant changes in dietary behaviors due to weight control or other reasons (e.g., culture and customs) before the survey; (b) previous cancer history (except glioma); (c) a history of hormonal drugs; (d) pregnant or lactating individuals; (e) neurological, digestive, endocrine, and other related diseases; and (f) a reported energy intake of more than 5000 or less than 400 kcal/day.

### 2.2. Dietary Intake Assessment

In the investigation, a reliable and effective food frequency questionnaire (FFQ) was used to evaluate dietary intake in the year before diagnosis [22,24]. The FFQ consisted of 114 items and allowed participants to report the average intake of various dietary items per specific time period, along with the frequency of consumption (daily, weekly, monthly, yearly, or never). The FFQ has been previously validated, and its reproducibility and validity were satisfactory [22]. To derive the average daily intake (in grams or milliliters), the consumption frequency was converted into a daily average intake frequency and multiplied by the average intake. Using the nutrient and energy data in the Chinese Food Composition Table [25], we calculated the average daily energy and nutrient intake.

Dietary surveys were conducted by uniformly trained and rigorously assessed investigators, and food picture pages containing various meal volumes and characteristics were used to help participants estimate their portion sizes in detail.

### 2.3. Assessment of Dietary Patterns Based on Priori Methods

The priori method was based on existing dietary guidelines or dietary patterns, to calculate the comprehensive score of the degree to which an individual’s diet met dietary recommendations. Based on the scores, the association between adherence to this dietary pattern and disease can be further evaluated. Each subject received a score for each dietary pattern. These dietary patterns were mainly a summary of dietary recommendations given by current epidemiological studies and nutritional knowledge for health or certain diseases. Thus, the dietary scores of priori methods measured the degree to which an individual adhered to these specific dietary patterns. Our investigation focused on five widely recognized dietary patterns: the Mediterranean diet, the Dietary Approaches to Stop Hypertension (DASH) diet, the Mediterranean-DASH diet Intervention for Neurodegenerative Delay (MIND diet), the Paleolithic diet, and the Planetary Health diet (PH diet). Supplementary Material A provides specific scoring details.

### 2.4. Assessment of Dietary Patterns Based on the Posterior Method

Principal component analysis (PCA) was utilized in the posterior method to isolate dietary factors. According to the type of food, the 114 items were divided into 21 food groups (Table S1). From the food intake, PCA was applied to extract dietary factors. The Kaiser–Meyer–Olkin (KMO) test and Bartlett Test of Sphericity were used to measure whether these dietary data applied to PCA. When the KMO score was more than 0.5 and the *p*-value of the Bartlett Test of Sphericity was under 0.05, these data were suitable for PCA. To make the data more comprehensible, orthogonal varimax rotation was performed after the principal components were extracted. The number of factors retained as the primary mode was determined by the eigenvalues (>1) and the gravel plot. Finally, the food group with an absolute value of factor load greater than 0.35 was considered to be the main contributor to the dietary factor (dietary pattern).

### 2.5. Covariates

The following variables, obtained through questionnaires, were considered to be potential confounding factors: basic personal information, lifestyle habits, and disease history. Basic personal information mainly included age, sex, occupation, education level, and household income. Lifestyle habits mainly include high-risk residential areas [26], smoking status, alcohol consumption, and physical activity. Based on existing questionnaires, metabolic equivalent was calculated to assess physical activity [27]. Disease histories included allergies, head trauma, and family cancer. Allergy history referred to whether the subject had allergic diseases. If the subject’s head had been injured before, it was considered as their history of head trauma. Family history of cancer referred to whether the relatives of the subject within three generations had cancer. In addition, study subjects‘ heights and weights were measured at the time of the investigation. Participants wore light indoor clothing, did not wear shoes, and remained on an empty stomach during the survey. Body mass index (BMI), which was computed as the square of body weight (kg) divided by height (m), was accurate to one decimal place for both weight and height.

### 2.6. Statistical Analysis

The basic characteristics of the glioma group and the healthy group were described, continuous variables were compared using the mean and standard deviation, and intergroup comparisons were made using the *t*-test. Categorical variables were reported as percentages, and comparisons across groups were made using the chi-square test. The association between the scores of each dietary pattern was evaluated using the Pearson correlation coefficient. Individuals were further divided into tertiles based on their scores for each dietary pattern or dietary factor. T1 represented the lowest tertile and T3 represented the highest tertile. ORs and 95% CIs for dietary pattern scores and risk of glioma were calculated via logistic regression. Model 1 was a simple logistic regression without adjustments to any confounding factors. Model 2 was a logistic regression model that adjusted for basic personal information (age, BMI, education level, occupation, and household income), lifestyle (high-risk residential areas, alcohol consumption, smoking status, and physical activity), disease status (history of head trauma, history of allergies, and family history of cancer), and energy intake. Among them, age, BMI, and energy intake were continuous variables. The others were categorical variables.

Subgroup analyses were conducted by some confounding variables. Sensitivity analysis was performed using the posterior method. The factors for each dietary pattern were determined, and Models 1 and 2 were used to determine the connection between dietary factors and gliomas.

Restricted cubic spline (RCS) analysis was used to determine the dose–response association between priori method scores and glioma. The reference value (OR = 1) was placed at the 10th percentile for each model, and four nodes were distributed across every 20 percentiles [28,29].

To further explore whether the association between diet and glioma was due to BMI. We used a causal mediating analysis to assess the mediating effect of BMI on the dietary pattern–glioma association [30]. We used dietary pattern score as an independent variable, BMI as a mediator, suffering from glioma as a dependent variable, and other variables as covariates to fit a mediating model. The “mediation” function in R was used to calculate the total effect, average causal mediation effects (ACMEs), and average direct effects (ADEs) between the two [31], as well as to estimate the proportion of BMI-mediated outcomes with significant indirect effects.

SPSS 26.0 and R software 4.1.1 were used for all statistical analyses. *p* < 0.05 indicated statistical significance for all bilateral statistical tests.

## 3. Results

### 3.1. Characteristics of the Study Population and Dietary Patterns

**Figure 1:**
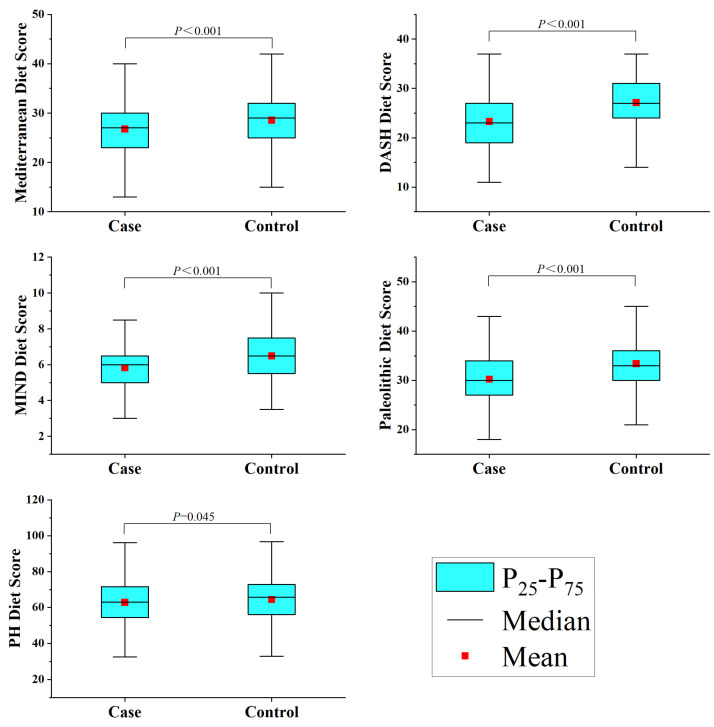
Dietary pattern scores among study participants.

This study included 1012 participants, with 506 cases in the glioma group. The glioma group was composed of 104 astrocytomas, 67 oligodendrogliomas, 237 glioblastomas, 18 diffuse midline gliomas, and 80 others. The study subject composition in different age groups mimicked that found in the general population in terms of alcohol consumption, history of allergies, high-risk residential area, physical activity, history of head trauma, education level, and household income (Table S2). However, the younger glioma case group had a higher BMI (*p* < 0.001) and a greater percentage of smokers (*p* = 0.017), though there were no notable changes in occupation (*p* = 0.058) or history of cancer (*p* = 0.814). The family members of the older group with gliomas had a higher rate of cancer (*p* < 0.001), but there were no notable changes in BMI (*p* = 0.347), occupation (*p* = 0.327), or smoking status (*p* = 0.285) (Table 1). Additionally, the healthy group had higher scores than the glioma group across all five dietary scores, as depicted in Figure 1.

### 3.2. Association between Dietary Pattern Score and Glioma

Table 2 displays the findings of the association between the five dietary patterns and gliomas. In Model 2, the third tertile scores for the Mediterranean diet (OR = 0.29; 95% CI: 0.17–0.52), the DASH diet (OR = 0.09; 95% CI: 0.04–0.18), the MIND diet (OR = 0.25; 95% CI: 0.14–0.44), and the Paleolithic diet (OR = 0.13; 95% CI: 0.06–0.25) were all associated with a reduced glioma risk compared to the first tertile. However, the PH diet did not exhibit a statistically significant association.

### 3.3. Dietary Pattern Score and Pathological Classification and Grade of Glioma

Table 3 showed that dietary patterns had different effects on different glioma subtypes. For astrocytoma, the Mediterranean diet (OR = 0.84; 95% CI: 0.72–0.99), the DASH diet (OR = 0.62; 95% CI: 0.45–0.85), the MIND diet (OR = 0.48; 95% CI: 0.27–0.86), and the Paleolithic diet (OR = 0.65; 95% CI: 0.48–0.88) were associated with a reduced risk. For glioblastoma, the Mediterranean diet (OR = 0.91; 95% CI: 0.84–0.99), the DASH diet (OR = 0.73; 95% CI: 0.62–0.85), the MIND diet (OR = 0.44; 95% CI: 0.27–0.72), and the Paleolithic diet (OR = 0.77; 95% CI: 0.67–0.88) were associated with a reduced risk. However, due to the limited sample size of oligodendroglioma, further analysis was not conducted.

The results of dietary pattern scores and various grades of glioma showed that the Mediterranean diet (OR = 0.85; 95% CI: 0.75–0.97), the DASH diet (OR = 0.63; 95% CI: 0.47–0.85), the MIND diet (OR = 0.60; 95% CI: 0.36–0.99), and the Paleolithic diet (OR = 0.72; 95% CI: 0.59–0.88) were associated with a reduced risk of low-grade glioma. For high-grade glioma, the Mediterranean diet (OR = 0.90; 95% CI: 0.84–0.96), the DASH diet (OR = 0.78; 95% CI: 0.70–0.86), the MIND diet (OR = 0.48; 95% CI: 0.34–0.69), and the Paleolithic diet (OR = 0.80; 95% CI: 0.73–0.88) were associated with a reduced risk (Table S3).

### 3.4. Posterior Method and Risk of Glioma

In this study, the KMO score was determined to be 0.771 and the Bartlett’s sphericity test score was *p* < 0.001, suggesting that data from these food groups could be analyzed by using the test. As shown in Table S4, six dietary factors were obtained via the PCA of 21 food groups. The first factor mainly included whole grains, legumes and products, tubers, vegetables, fungi and algae, fruits, fish and seafood, dairy products, nuts, and sweet food. The second factor mainly included refined grains, red meat, salt, oils, and alcohol. The third factor mainly included tubers, poultry, and dairy products. The fourth factor mainly included animal viscera, tea and coffee, and alcohol. The fifth factor mainly included eggs, sugary drinks, tea and coffee, and cured and processed products. The sixth factor mainly included refined grains and alcohol. These components accounted for 52.14% of the variance in the initial dataset.

Table 4 presents the associations between the six dietary factors and gliomas. In Model 2, after adjusting for other variables, the results indicated that compared to the first tertile, dietary factor 1 (OR = 0.03; 95% CI: 0.01–0.08), dietary factor 3 (OR = 0.44; 95% CI: 0.26–0.77), and dietary factor 4 (OR = 0.41; 95% CI: 0.23–0.74) were significantly associated with a reduced glioma risk. Dietary factor 2 (OR = 4.99; 95% CI: 2.56–9.71) and dietary factor 6 (OR = 3.75; 95% CI: 1.89–7.44) were significantly associated with an elevated glioma risk. However, dietary factor 5 was not statistically significant.

### 3.5. Subgroup Analysis

After stratification by various factors and adjusting for relevant covariates using Model 2, the results for most subgroups of the five dietary patterns were in accordance with the general population. However, the results for individual subgroups were constrained by the small sample (Table S5).

### 3.6. Dose–Response Relationship

**Figure 2:**
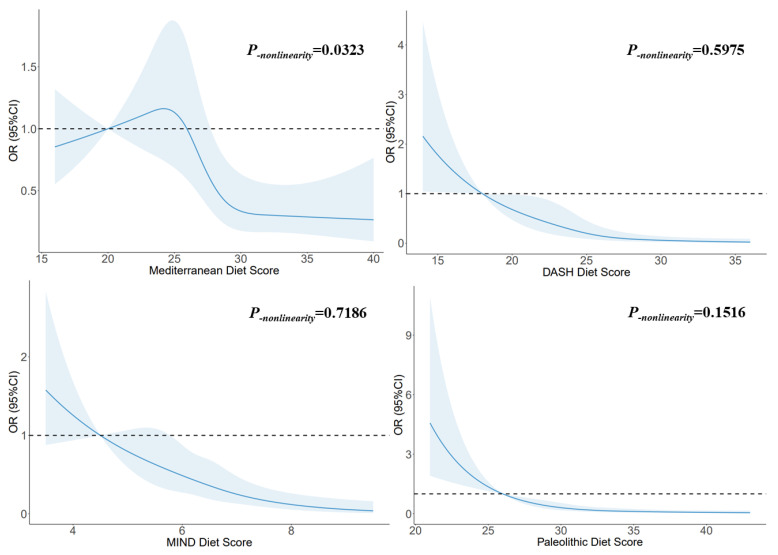
The RCS for the associations between dietary pattern scores and glioma. The lines represent adjusted odds ratios based on RCSs for the dietary pattern scores in the regression model. Knots were placed at the 20th, 40th, 60th, and 80th percentiles of the dietary pattern scores, and the reference value was set at the 10th percentile. The adjusted factors were the same as in Model 2.

Figure 2 illustrates the utilization of RCSs to flexibly model and visually represent the relationship between glioma and dietary pattern scores. A non-linear dose–response relationship was observed between the Mediterranean diet score and glioma (*p_-nonlinearity_* = 0.0323), and the risk decreased with increasing score when the scores exceeded 27. A linear dose–response relationship was observed between the DASH diet score and glioma (*p_-nonlinearity_* = 0.5975), and the risk decreased with increasing score when the score exceeded 18. A linear dose–response relationship was observed between the MIND diet score and glioma (*p_-nonlinearity_* = 0.7186), and the risk decreased with increasing score. A linear dose–response relationship was observed between the Paleolithic diet scores and glioma (*p_-nonlinearity_* = 0.1516), and the risk decreased with increasing score. When the score exceeded 30, the trend tended to be stable.

### 3.7. Mediating Effect Based on BMI

The mediating effects suggested that the DASH diet was associated with a reduction in glioma risk due to a decrease in BMI (2.82%, *p* = 0.014), and the MIND diet was associated with a reduction in glioma risk due to a decrease in BMI (3.24%, *p* = 0.008). The effects of the Mediterranean diet and Paleolithic diet on glioma were independent of BMI (Table S6).

## 4. Discussion

Our research evaluated the association between glioma and five dietary patterns in a Chinese population. The Mediterranean diet, the DASH diet, the MIND diet, and the Paleolithic diet were all negatively associated with glioma risk. Similar results were discovered in gliomas of various pathological subgroups and grades. The dose–response relationships depicted in the RCS further confirmed the significant linear or nonlinear dose–response relationships between these dietary patterns and glioma. This association of dietary factors with glioma in posterior methods was similar to the results for dietary patterns in the priori method. In addition, subgroup analyses showed that the results of this study were relatively robust.

For a long time, the Mediterranean diet was considered to reduce the incidence of many chronic diseases and prolong life [32]. The Mediterranean diet and cancer (such as overall cancer risk [33], lung cancer [34], colon cancer, and gastric cancer [35]) have been associated in a number of earlier studies,. However, studies of the Mediterranean diet and gliomas are rare. For glioma, our research revealed that following the Mediterranean diet was associated with a reduced risk (OR = 0.92; 95% CI: 0.88–0.96). This is similar to the viewpoint of Mousavi et al. in Iran, who found a similar association between this dietary pattern and glioma by comparing the Mediterranean diet in 128 patients with glioma and 256 healthy people (OR = 0.36; 95% CI: 0.16–0.78) [20]. However, Kuan et al. observed opposite results in a cohort study (relative risk (RR) = 1.24; 95% CI: 1.05–1.46), and this association became insignificant after removing the earliest five years of data (RR = 1.17; 95% CI: 0.95–1.45). This was considered to be related to dietary modifications brought on by preclinical glioma [21]. However, the mechanism of the Mediterranean diet for gliomas was unclear. It was considered that the food groups in the Mediterranean diet provided various types of polyphenols and a large number of antioxidants, such as resveratrol, tannic acid, etc. [36]. These high levels of antioxidants and anti-inflammatory nutrients have inhibitory effects against the proliferation of cancer cells [37,38]. Cell experiments have shown that resveratrol could play an antiproliferative effect as an adenosine receptor agonist in glioma cells by regulating the tumor microenvironment [39]. Animal experiments have also found that when rats with glioblastoma were fed by gavage with tannic acid for half a month, it significantly reduced the levels of reactive oxygen species and thiobarbiturate reactants in brain tissue and restored superoxide dismutase activity, suggesting that tannic acid can antagonize glioblastoma by regulating oxidative stress levels [40]. Phytochemicals such as lignans [41] and quercetin [42] have shown similar results. One of the main sources of these substances is wine [43], and moderate drinking of wine is the typical Mediterranean diet’s main component [38,44]. This was hardly present in other dietary patterns.

The DASH diet was originally used to prevent cardiovascular diseases such as hypertension; however, in recent years it has also been found to be very closely related to cancer [45,46]. Benisi-Kohansal et al. reported that people who followed the DASH diet in the Middle East had a 72% lower glioma risk than those who followed the lowest (OR = 0.28; 95% CI 0.13–0.57) [47]. This was very similar to our findings (OR = 0.80; 95% CI: 0.74–0.85). However, in the study of Kuan et al., the results of the DASH diet were comparable to those of the Mediterranean diet; that is, the association with glioma was not significant [21]. The DASH diet promoted vegetables and fruits and restricted red and processed meats, which was highly consistent with dietary factor 1 (OR = 0.03; 95% CI: 0.01–0.08) and dietary factor 2 (OR = 4.99; 95% CI: 2.56–9.71) extracted by PCA in this study, indicating the DASH diet’s possible mechanism for lowering glioma risk. On the one hand, vegetables and fruits are rich in a large number of anti-tumor substances, such as flavonoids, glucosinolate, and vitamins; these compounds are involved in DNA damage repair, methylation regulation, and the promotion of apoptosis [48], and they play a certain limiting role in the occurrence of glioma [49,50]. On the other hand, processed meat products are rich in N-nitroso compounds and their precursor substances during processing and preservation [51]. Studies have shown that N-nitroso compounds have inducing effects on a variety of cancers [52]. N-nitroso compounds can cause gliomas by impairing the effectiveness of DNA damage repair, according to animal studies [53]. In addition, the development, invasion, and growth of gliomas are all significantly influenced by inflammation [54,55,56]. The DASH diet can maintain a lower level of C-reactive protein in the cycle, exert anti-inflammatory effects, and thus prevent the development of glioma [57,58].

The MIND diet mainly provides a protective effect for cognitive functions by aggregating foods that have benefits for the brain and central nervous systems, such as whole grains, legumes, nuts, olive oil, etc., found in the Mediterranean diet and the DASH diet [59]. Since the purpose of this dietary pattern is to prevent neurodegenerative diseases, the majority of studies have concentrated on the senior population’s cognitive health [60]. Thus, there has been very little study on the MIND diet and cancer. There are currently only two breast cancer studies [61,62] and one glioma study [63] that show a preventive effect against cancer. Our results also show the MIND diet’s protection against gliomas (OR = 0.55; 95% CI: 0.44–0.68), and its effect was somewhere between those of the DASH diet and the Mediterranean diet, because neurodegenerative diseases are closely associated with gliomas. Lehrer discovered a strong association between the mortality from malignant brain tumors and Alzheimer’s disease in the United States between 1999 and 2016 (*p* < 0.001) [64]. Thus, in addition to mechanisms similar to those of the other diets, the MIND diet may indirectly reduce the incidence of glioma by protecting cognitive function.

The Paleolithic diet is based on the lifestyle of humans in the Paleolithic Period and advocates a diet pattern with unprocessed animal and plant food as the main food [65]. In recent years, it has been demonstrated that this dietary pattern effectively protects against chronic disease [66]. Although Whalen et al. found in a cohort study based on the U.S. population that adherence to the Paleolithic diet pattern significantly reduced cancer mortality (hazard ratio (HR) = 0.72; 95% CI: 0.55–0.95) [67], there are still few studies on this diet pattern and cancer. Our study represents the first exploration of the effects of the Paleolithic diet on gliomas, and we describe a significant linear dose–response relationship between them (*p_-nonlinearity_* = 0.1516). Similar results have at present only been reported for breast cancer [68]. Furthermore, there is some controversy about this dietary pattern. Compared to other dietary patterns, it is the only one that does not limit meat intake. However, we believe that this is not contradictory to other dietary patterns. It emphasizes lean meat more than processed meats and animal fats [65]. In addition, it restricts cereals, beans, and dairy products, which are frequently regarded as healthy foods; however, it seems to be more helpful in limiting the intake of added sugars and fats [69]. Because the Paleolithic diet might reduce levels of oxidative stress and inflammation throughout the body, we still believe that it may help prevent cancer including gliomas [70,71].

The PH diet is a recommended environmentally friendly and health-conscious eating model proposed by the EAT-Lancet Commission. Unlike the goals of other dietary patterns, this dietary pattern aims to maintain human health in the face of environmental degradation caused by food production, so that the environment and human health can be jointly sustained [72]. The results of a German cohort study have shown that this dietary pattern significantly reduces greenhouse gas emissions (β = −0.22; 95% CI: −0.30–−0.14) and land use (β = −0.40; 95% CI: −0.52–−0.29) and also has a certain control effect on BMI [73]. However, our study did not find that the PH diet had any effects against glioma (OR = 0.99; 95% CI: 0.97–1.01). This may be related to lower adherence to this dietary pattern. The PH diet scores range from 0 to 150 points [74], but healthy people scored less than half of their highest score in this study (64.45 ± 13.06). Similar results were found in the Brazilian population, whose average overall score was only 45.9 (95% CI: 45.6–46.1) [75]. This dietary pattern has more refined requirements for food intake (including adequacy, optimum, ratio, and moderation) [74] and is closely related to economic income [76]; this made it more difficult for people to adhere to this dietary pattern.

The results of PCA suggested that increasing the intake of plant-based foods (vegetables, fruits, and whole grains) and fish and limiting foods rich in carbohydrates, fats, and salts (refined grains, meats, and processed foods) may reduce glioma risk. This matches the findings of an earlier investigation analyzing the association of nutrient intake patterns with gliomas [77]; because most of those dietary factors were in accordance with the components of the four dietary patterns previously described, they mutually confirm the impact of dietary patterns on glioma. In addition, previous studies have reported a significant impact of BMI on glioma, and healthy dietary habits are closely related to BMI [78]. We also further explore whether these dietary patterns might have an effect on gliomas by affecting BMI. According to the results with mediating effect, BMI only plays a small mediating role (about 3%) in the effects of the DASH diet and the MIND diet on glioma, and most diets still have direct effects on glioma. These potential mechanisms should be further explored in subsequent studies.

In addition, although no such dietary patterns and glioma prognoses have been reported, ketogenic diets with similar components to these healthy eating patterns have long been thought to aid in the treatment of glioma. Rieger et al. found that the median progression-free survival of all patients was 5 (range: 3–13) weeks and the median survival after enrollment was 32 weeks in 20 patients with recurrent glioblastoma who underwent ketogenic diet intervention [79]. Porper et al. found better median progression-free survival for newly diagnosed and relapsed diseases of 10 months and 4 months, respectively, in patients with gliomas treated on ketogenic diets in combination with metformin [80]. These all suggest that a healthy dietary pattern is also closely related to the prognosis of glioma.

This study represents the first investigation conducted in China to explore the connection between dietary patterns and glioma. Consequently, this study has some strengths. Firstly, we fully investigated the relationship between gliomas and five popular dietary patterns and made a detailed analysis based on the pathological classification and grading of gliomas. The complex relationships between different foods in this study were considered as a whole, reflecting the actual eating habits of individuals, and providing more information to illustrate the association between diet and glioma by describing the dose–response relationship. Second, this study also explored dietary patterns through PCA based on the posterior method. Using both methods (priori and posterior) on the same data yielded similar results. This allowed us to assess the diets of study subjects extensively, both avoiding some of the limitations associated with subjectivity in creating food groups and choosing which factors to retain, and providing specific information on multiple foods in the priori method [81]. Moreover, the study was the largest Chinese population study on dietary patterns and glioma in the past two decades, and its results provide some support for the creation of primary glioma prevention measures. However, the study still has some limitations. The recall bias was inevitable in a case-control study; however, we included as many new cases as possible, with most cases taking less than 3 months from diagnosis to investigation. For choice bias, we also used individual matching of age and sex, which minimized this bias. Second, due to sample size limitations, this study could not further adjust for other potential confounding factors such as sleep, stress, and other factors. Considering the large content of the dietary questionnaire, no more confounding factors were investigated to ensure good compliance; however, in this study, the potential confounding factors have been adjusted as much as possible, including history of disease and residential environment. Finally, since it was not a prospective study, this study was not able to determine causality between glioma and diet.

## 5. Conclusions

To summarize, our study findings indicate that, for the Chinese population, adhering to the Mediterranean diet, the DASH diet, the MIND diet, or the Paleolithic diet is associated with a reduced risk of glioma. Furthermore, we observed significant dose–response relationships, indicating that higher adherence to these dietary patterns has associations with a further-reduced glioma risk. However, it is worth noting that our study is observational, and thus causality cannot be definitively established. Future studies should focus on conducting prospective research to verify the causal relationship between these dietary patterns and glioma risk.

## Figures and Tables

**Table 1:** Basic characteristics of the study participants in different age groups.

	Age ≤ 40 (*n* = 500)			Age > 40 (*n* = 512)		
	Case (*n* = 239)	Control (*n* = 261)	*p* ^a^	Case (*n* = 267)	Control (*n* = 245)	*p* ^a^
Age (years)	31.10 ± 6.14	30.70 ± 5.10	0.427	52.93 ± 8.11	52.28 ± 8.53	0.379
Sex, *n* (%)			0.880			0.885
Male	55.6	56.3		56.6	55.9	
Female	44.4	43.7		43.4	44.1	
BMI (kg/m^2^)	24.01 ± 3.63	22.35 ± 3.40	<0.001	24.04 ± 2.88	23.80 ± 2.96	0.347
High-risk residential area, (%)			0.051			0.295
Yes	23.4	16.5		19.5	23.3	
No	76.6	83.5		80.5	76.7	
Occupation, (%)			0.058			0.327
Manual workers	20.1	12.3		32.2	29.0	
Mental workers	71.5	78.1		35.2	41.6	
Others	8.4	9.6		32.6	29.4	
Education level, (%)			<0.001			<0.001
Primary school and below	1.3	0.8		12.0	4.5	
Middle school	31.0	11.1		50.9	40.0	
University and above	67.7	88.1		37.1	55.5	
Household income, (%)			<0.001			<0.001
<3000 ¥/month	5.4	14.6		13.5	22.0	
3000–10,000 ¥/month	78.7	52.1		73.4	46.2	
>10,000 ¥/month	15.9	33.3		13.1	31.8	
Smoking status, (%)			0.017			0.285
Never	72.0	81.6		68.2	68.5	
Former smoker	7.5	3.1		17.6	13.5	
Current smoker	20.5	15.3		14.2	18.0	
Alcohol consumption, (%)			<0.001			<0.001
Never	68.2	56.3		60.3	56.3	
Occasional drinker	13.8	32.6		12.0	27.8	
Frequent drinker	18.0	11.1		27.7	15.9	
History of allergies, (%)			0.005			0.037
Yes	7.9	16.1		7.5	13.1	
No	92.1	83.9		92.5	86.9	
History of head trauma, (%)			0.923			0.269
Yes	10.5	10.7		12.0	9.0	
No	89.5	89.3		88.0	91.0	
Family history of cancer, (%)			0.814			<0.001
Yes	24.3	23.4		35.2	18.8	
No	75.7	76.6		64.8	81.2	
Physical activity, (%)			<0.001			<0.001
Low	15.9	48.7		11.6	42.8	
Moderate	45.2	33.7		37.8	39.2	
Extreme	38.9	17.6		50.6	18.0	

^a.^ *p*-values were derived from the Student’s *t*-test for continuous variables according to the data distribution and the chi-square test for categorical variables.

**Table 2:** Adjusted ORs and 95% CIs for the association between dietary pattern scores and glioma.

	T1	T2	T3	Continuous ^c^	*p_-continuous_*
Mediterranean Diet	≤26	27–30	>30		
Case/Control	245/166	144/151	117/189		
Model 1 ^a^	1	0.61 (0.45–0.84)	0.43 (0.31–0.58)	0.94 (0.92–0.96)	<0.001
Model 2 ^b^	1	0.41 (0.23–0.74)	0.29 (0.17–0.52)	0.92 (0.88–0.96)	<0.001
DASH Diet	≤23	24–28	>28		
Case/Control	268/117	160/183	78/206		
Model 1 ^a^	1	0.35 (0.25–0.50)	0.15 (0.10–0.23)	0.84 (0.82–0.87)	<0.001
Model 2 ^b^	1	0.30 (0.16–0.56)	0.09 (0.04–0.18)	0.80 (0.74–0.85)	<0.001
MIND	≤5.5	6–6.5	>6.5		
Case/Control	249/142	146/150	111/214		
Model 1 ^a^	1	0.57 (0.41–0.78)	0.28 (0.20–0.40)	0.64 (0.57–0.71)	<0.001
Model 2 ^b^	1	0.48 (0.28–0.85)	0.25 (0.14–0.44)	0.55 (0.44–0.68)	<0.001
Paleolithic Diet	≤30	31–34	>34		
Case/Control	271/131	141/163	94/212		
Model 1 ^a^	1	0.35 (0.25–0.50)	0.20 (0.14–0.28)	0.85 (0.83–0.88)	<0.001
Model 2 ^b^	1	0.31 (0.16–0.58)	0.13 (0.06–0.25)	0.82 (0.77–0.87)	<0.001
PH Diet	≤58.61	58.61–69.64	>69.64		
Case/Control	182/156	171/166	153/184		
Model 1 ^a^	1	0.87 (0.64–1.18)	0.70 (0.51–0.95)	0.99 (0.98–1.00)	0.035
Model 2 ^b^	1	0.94 (0.55–1.60)	0.61 (0.35–1.08)	0.99 (0.97–1.01)	0.198

Note: T1, T2, and T3 represent the tertiles of each dietary pattern score. ^a^ Model 1: unadjusted model. ^b^ Model 2: adjusted for age, BMI, occupation, education level, household income, high-risk residential areas, smoking status, alcohol consumption, history of allergies, history of head trauma, family history of cancer, physical activity, and energy intake. ^c^ The results were obtained from dietary scores as continuous variables.

**Table 3:** Adjusted ORs and 95% CIs for the association between dietary pattern scores and glioma of different pathological classifications.

Pathological Classification ^a^	Model 1 ^b^	*p*	Model 2 ^c^	*p*
Astrocytoma				
Mediterranean Diet	0.91 (0.86–0.97)	0.005	0.84 (0.72–0.99)	0.031
DASH Diet	0.79 (0.71–0.87)	<0.001	0.62 (0.45–0.85)	0.003
MIND Diet	0.64 (0.50–0.83)	0.001	0.48 (0.27–0.86)	0.013
Paleolithic Diet	0.84 (0.78–0.91)	<0.001	0.65 (0.48–0.88)	0.006
PH Diet	0.99 (0.97–1.01)	0.466	1.01 (0.97–1.05)	0.794
Glioblastoma				
Mediterranean Diet	0.94 (0.91–0.98)	0.001	0.91 (0.84–0.99)	0.028
DASH Diet	0.84 (0.80–0.88)	<0.001	0.73 (0.62–0.85)	<0.001
MIND Diet	0.65 (0.55–0.77)	<0.001	0.44 (0.27–0.72)	0.001
Paleolithic Diet	0.86 (0.82–0.90)	<0.001	0.77 (0.67–0.88)	<0.001
PH Diet	0.99 (0.98–1.01)	0.265	1.00 (0.97–1.04)	0.990

Note: Due to the small sample size of oligodendroglioma, no further analysis was conducted. ^a^ The results were derived from dietary scores expressed as continuous variables. ^b^ Model 1: unadjusted model. ^c^ Model 2: adjusted for age, BMI, occupation, education level, household income, high-risk residential areas, smoking status, alcohol consumption, history of allergies, history of head trauma, family history of cancer, physical activity, and energy intake.

**Table 4:** Adjusted ORs and 95% CIs for the association between dietary factors in PCA and glioma.

	T1	T2	T3	*p_-trend_*
Factor 1	≤−0.46	−0.46–0.94	>0.94	
Case/Control	328/169	123/169	55/168	
Model 1 ^a^	1	0.34 (0.24–0.48)	0.17 (0.11–0.25)	<0.001
Model 2 ^b^	1	0.16 (0.08–0.30)	0.03 (0.01–0.08)	<0.001
Factor 2	≤−0.94	−0.94–−0.26	>−0.26	
Case/Control	85/171	81/167	340/168	
Model 1 ^a^	1	0.98 (0.65–1.46)	3.97 (2.79–5.64)	<0.001
Model 2 ^b^	1	1.27 (0.66–2.46)	4.99 (2.56–9.71)	<0.001
Factor 3	≤−0.12	−0.12–0.33	>0.33	
Case/Control	201/171	181/169	124/166	
Model 1 ^a^	1	0.87 (0.64–1.18)	0.60 (0.43–0.84)	0.005
Model 2 ^b^	1	0.57 (0.34–0.95)	0.44 (0.26–0.77)	0.003
Factor 4	≤−0.40	−0.40–0.01	>0.01	
Case/Control	246/178	114/161	146/167	
Model 1 ^a^	1	0.50 (0.36–0.69)	0.60 (0.44–0.82)	0.014
Model 2 ^b^	1	0.37 (0.21–0.63)	0.41 (0.23–0.74)	0.018
Factor 5	≤−0.35	−0.35–0.25	>0.25	
Case/Control	136/171	192/167	178/168	
Model 1 ^a^	1	1.48 (1.08–2.02)	1.37 (0.99–1.89)	0.062
Model 2 ^b^	1	1.33 (0.79–2.25)	0.93 (0.55–1.56)	0.709
Factor 6	≤−0.38	−0.38–−0.08	>−0.08	
Case/Control	64/171	161/167	281/168	
Model 1 ^a^	1	2.92 (1.96–4.37)	5.42 (3.61–8.13)	<0.001
Model 2 ^b^	1	2.99 (1.63–5.47)	3.75 (1.89–7.44)	0.001

Note: T1, T2, and T3 represent the tertiles of each dietary factor score. ^a^ Model 1: unadjusted model. ^b^ Model 2: adjusted for age, BMI, occupation, education level, household income, high-risk residential areas, smoking status, history of allergies, history of head trauma, family history of cancer, physical activity, and energy intake.

## Data Availability

The data presented in this study are available on request from the corresponding author.

## Supplementary Materials

The following supporting information can be downloaded at https://www.mdpi.com/article/10.3390/nu15234886/s1: Supplementary Material A: Supplemental methods: components and scoring of the dietary pattern scores. Supplementary Material B: Table S1: Dietary items in the food frequency questionnaire; Table S2: Basic characteristics of all study participants; Table S3: Adjusted ORs and 95% CIs for the association between dietary pattern scores and glioma of different grades; Table S4: Factor loading matrix for the major dietary patterns derived from PCA; Table S5: Adjusted ORs and 95% CIs for gliomas in subgroups; Table S6: Dietary patterns’ influence on the proportion of gliomas, mediated by BMI; Figure S1: Flow chart of the case-control population matching. References [60,66,72,74,82,83,84] are cited in the supplementary materials.

## References

[1] Ostrom Q.T., Price M., Ryan K., Edelson J., Neff C., Cioffi G., Waite K.A., Kruchko C., Barnholtz-Sloan J.S. (2022). CBTRUS Statistical Report: Pediatric Brain Tumor Foundation Childhood and Adolescent Primary Brain and Other Central Nervous System Tumors Diagnosed in the United States in 2014–2018. Neuro-Oncology.

[2] Miller K.D., Ostrom Q.T., Kruchko C., Patil N., Tihan T., Cioffi G., Fuchs H.E., Waite K.A., Jemal A., Siegel R.L. (2021). Brain and other central nervous system tumor statistics, 2021. CA-Cancer J. Clin..

[3] Bellail A.C., Hunter S.B., Brat D.J., Tan C., Van Meir E.G. (2004). Microregional extracellular matrix heterogeneity in brain modulates glioma cell invasion. Int. J. Biochem. Cell Biol..

[4] Walsh K.M., Claus E.B. (2019). Diet and risk of glioma: Targets for prevention remain elusive. Neuro-Oncology.

[5] Clinton S.K., Giovannucci E.L., Hursting S.D. (2020). The World Cancer Research Fund/American Institute for Cancer Research Third Expert Report on Diet, Nutrition, Physical Activity, and Cancer: Impact and Future Directions. J. Nutr..

[6] Mayne S.T., Playdon M.C., Rock C.L. (2016). Diet, nutrition, and cancer: Past, present and future. Nat. Rev. Clin. Oncol..

[7] Aune D., Giovannucci E., Boffetta P., Fadnes L.T., Keum N., Norat T., Greenwood D.C., Riboli E., Vatten L.J., Tonstad S. (2017). Fruit and vegetable intake and the risk of cardiovascular disease, total cancer and all-cause mortality—A systematic review and dose-response meta-analysis of prospective studies. Int. J. Epidemiol..

[8] Nagao M., Tsugane S. (2016). Cancer in Japan: Prevalence, prevention and the role of heterocyclic amines in human carcinogenesis. Genes Environ..

[9] Bielecka J., Markiewicz-Zukowska R. (2020). The Influence of Nutritional and Lifestyle Factors on Glioma Incidence. Nutrients.

[10] Terry M.B., Howe G., Pogoda J.M., Zhang F.F., Ahlbom A., Choi W., Giles G.G., Little J., Lubin F., Menegoz F. (2009). An international case-control study of adult diet and brain tumor risk: A histology-specific analysis by food group. Ann. Epidemiol..

[11] Lee M., Wrensch M., Miike R. (1997). Dietary and tobacco risk factors for adult onset glioma in the San Francisco Bay Area (California, USA). Cancer Cause Control.

[12] Shahrestani M.A., Saneei P., Shayanfar M., Mohammad-Shirazi M., Sharifi G., Sadeghi O., Esmaillzadeh A. (2021). The relationship between rice consumption and glioma: A case-control study in adults. Sci. Rep..

[13] Song Y., Wang Z., Jin Y., Guo J. (2019). Association between tea and coffee consumption and brain cancer risk: An updated meta-analysis. World J. Surg. Oncol..

[14] Zhang W., Jiang J., Li X., He Y., Chen F., Li W. (2022). Dietary Factors and Risk of Glioma in Adults: A Systematic Review and Dose-Response Meta-Analysis of Observational Studies. Front. Nutr..

[15] Dianatinasab M., Wesselius A., Salehi-Abargouei A., Yu E., Brinkman M., Fararouei M., van den Brandt P., White E., Weiderpass E., Le Calvez-Kelm F. (2020). Adherence to a Western dietary pattern and risk of bladder cancer: A pooled analysis of 13 cohort studies of the Bladder Cancer Epidemiology and Nutritional Determinants international study. Int. J. Cancer.

[16] Hu F.B. (2002). Dietary pattern analysis: A new direction in nutritional epidemiology. Curr. Opin. Lipidol..

[17] Mahamat-Saleh Y., Cervenka I., Al R.M., Savoye I., Mancini F.R., Trichopoulou A., Boutron-Ruault M.C., Kvaskoff M. (2019). Mediterranean dietary pattern and skin cancer risk: A prospective cohort study in French women. Am. J. Clin. Nutr..

[18] Gioxari A., Tzanos D., Kostara C., Papandreou P., Mountzios G., Skouroliakou M. (2021). Mediterranean Diet Implementation to Protect against Advanced Lung Cancer Index (ALI) Rise: Study Design and Preliminary Results of a Randomised Controlled Trial. Int. J. Env. Res. Pub. Health.

[19] Kim J.H., Lee J., Choi I.J., Kim Y.I., Kim J. (2021). Dietary patterns and gastric cancer risk in a Korean population: A case-control study. Eur. J. Nutr..

[20] Mousavi S.M., Shayanfar M., Rigi S., Mohammad-Shirazi M., Sharifi G., Esmaillzadeh A. (2021). Adherence to the Mediterranean dietary pattern in relation to glioma: A case-control study. Clin. Nutr..

[21] Kuan A.S., Green J., Kitahara C.M., Berrington D.G.A., Key T., Reeves G.K., Floud S., Balkwill A., Bradbury K., Liao L.M. (2019). Diet and risk of glioma: Combined analysis of 3 large prospective studies in the UK and USA. Neuro-Oncology.

[22] Zhang W., He Y., Kang X., Wang C., Chen F., Kang Z., Yang S., Zhang R., Peng Y., Li W. (2023). Association between dietary minerals and glioma: A case-control study based on Chinese population. Front. Nutr..

[23] Louis D.N., Perry A., Wesseling P., Brat D.J., Cree I.A., Figarella-Branger D., Hawkins C., Ng H.K., Pfister S.M., Reifenberger G. (2021). The 2021 WHO Classification of Tumors of the Central Nervous System: A summary. Neuro-Oncology.

[24] Zhao W.-H., Huang Z.P., Zhang X., He L., Willett W., Wang J.-L., Hasegawa K., Chen J.-S. (2010). Reproducibility and Validity of a Chinese Food Frequency Questionnaire. Biomed. Environ. Sci..

[25] Yang Y.X. (2018). China Food Composition Tables.

[26] Morgan L.L., Miller A.B., Sasco A., Davis D.L. (2015). Mobile phone radiation causes brain tumors and should be classified as a probable human carcinogen (2A) (review). Int. J. Oncol..

[27] Craig C.L., Marshall A.L., Sjostrom M., Bauman A.E., Booth M.L., Ainsworth B.E., Pratt M., Ekelund U., Yngve A., Sallis J.F. (2003). International physical activity questionnaire: 12-country reliability and validity. Med. Sci. Sports Exer..

[28] Zhang W., Du J., Li H., Yang Y., Cai C., Gao Q., Xing Y., Shao B., Li G. (2020). Multiple-element exposure and metabolic syndrome in Chinese adults: A case-control study based on the Beijing population health cohort. Environ. Int..

[29] Moon K.A., Guallar E., Umans J.G., Devereux R.B., Best L.G., Francesconi K.A., Goessler W., Pollak J., Silbergeld E.K., Howard B.V. (2013). Association between exposure to low to moderate arsenic levels and incident cardiovascular disease. A prospective cohort study. Ann. Intern. Med..

[30] Imai K., Keele L., Tingley D. (2010). A general approach to causal mediation analysis. Psychol. Methods.

[31] Valente M.J., Rijnhart J., Smyth H.L., Muniz F.B., MacKinnon D.P. (2020). Causal Mediation Programs in R, Mplus, SAS, SPSS, and Stata. Struct. Equ. Model..

[32] Tosti V., Bertozzi B., Fontana L. (2018). Health Benefits of the Mediterranean Diet: Metabolic and Molecular Mechanisms. J. Gerontol. A-Biol..

[33] Schulpen M., van den Brandt P.A. (2021). Adherence to the Mediterranean Diet and Overall Cancer Incidence: The Netherlands Cohort Study. J. Acad. Nutr. Diet..

[34] Krusinska B., Hawrysz I., Wadolowska L., Slowinska M.A., Biernacki M., Czerwinska A., Golota J.J. (2018). Associations of Mediterranean Diet and a Posteriori Derived Dietary Patterns with Breast and Lung Cancer Risk: A Case-Control Study. Nutrients.

[35] Schwingshackl L., Schwedhelm C., Galbete C., Hoffmann G. (2017). Adherence to Mediterranean Diet and Risk of Cancer: An Updated Systematic Review and Meta-Analysis. Nutrients.

[36] Naska A., Trichopoulou A. (2014). Back to the future: The Mediterranean diet paradigm. Nutr. Metab. Cardiovas..

[37] Emma M.R., Augello G., Di Stefano V., Azzolina A., Giannitrapani L., Montalto G., Cervello M., Cusimano A. (2021). Potential Uses of Olive Oil Secoiridoids for the Prevention and Treatment of Cancer: A Narrative Review of Preclinical Studies. Int. J. Mol. Sci..

[38] Mentella M.C., Scaldaferri F., Ricci C., Gasbarrini A., Miggiano G. (2019). Cancer and Mediterranean Diet: A Review. Nutrients.

[39] Sanchez-Melgar A., Munoz-Lopez S., Albasanz J.L., Martin M. (2021). Antitumoral Action of Resveratrol through Adenosinergic Signaling in C6 Glioma Cells. Front. Neurosci..

[40] Bona N.P., Soares M., Pedra N.S., Spohr L., Da S.D.S.F., de Farias A.S., Alvez F.L., de Moraes M.B., Luduvico K.P., Spanevello R.M. (2022). Tannic Acid Attenuates Peripheral and Brain Changes in a Preclinical Rat Model of Glioblastoma by Modulating Oxidative Stress and Purinergic Signaling. Neurochem. Res..

[41] Maimaitili A., Shu Z., Cheng X., Kaheerman K., Sikandeer A., Li W. (2017). Arctigenin, a natural lignan compound, induces G0/G1 cell cycle arrest and apoptosis in human glioma cells. Oncol. Lett..

[42] Tamtaji O.R., Razavi Z.S., Razzaghi N., Aschner M., Barati E., Mirzaei H. (2022). Quercetin and Glioma: Which Signaling Pathways are Involved?. Curr. Mol. Pharmacol..

[43] Amor S., Chalons P., Aires V., Delmas D. (2018). Polyphenol Extracts from Red Wine and Grapevine: Potential Effects on Cancers. Diseases.

[44] Trichopoulou A., Critselis E. (2004). Mediterranean diet and longevity. Eur. J. Cancer Prev..

[45] Soltani S., Arablou T., Jayedi A., Salehi-Abargouei A. (2020). Adherence to the dietary approaches to stop hypertension (DASH) diet in relation to all-cause and cause-specific mortality: A systematic review and dose-response meta-analysis of prospective cohort studies. Nutr. J..

[46] Toorang F., Sasanfar B., Hadji M., Esmaillzadeh A., Zendehdel K. (2020). Adherence to “dietary approaches to stop hypertension” eating plan in relation to gastric cancer. Nutr. J..

[47] Benisi-Kohansal S., Shayanfar M., Mohammad-Shirazi M., Tabibi H., Sharifi G., Saneei P., Esmaillzadeh A. (2016). Adherence to the Dietary Approaches to Stop Hypertension-style diet in relation to glioma: A case-control study. Brit. J. Nutr..

[48] McCullough M.L., Giovannucci E.L. (2004). Diet and cancer prevention. Oncogene.

[49] Hung C.F., Lu K.H. (2001). Vitamin C inhibited DNA adduct formation and arylamine N-acetyltransferase activity and gene expression in rat glial tumor cells. Neurochem. Res..

[50] Hervouet E., Debien E., Campion L., Charbord J., Menanteau J., Vallette F.M., Cartron P.F. (2009). Folate supplementation limits the aggressiveness of glioma via the remethylation of DNA repeats element and genes governing apoptosis and proliferation. Clin. Cancer Res..

[51] Lijinsky W. (1999). N-Nitroso compounds in the diet. Mutat. Res.-Genet. Toxicol. Environ. Mutagen..

[52] Mirvish S.S. (1995). Role of N-nitroso compounds (NOC) and N-nitrosation in etiology of gastric, esophageal, nasopharyngeal and bladder cancer and contribution to cancer of known exposures to NOC. Cancer Lett..

[53] Goth R., Rajewsky M.F. (1974). Persistence of O6-ethylguanine in rat-brain DNA: Correlation with nervous system-specific carcinogenesis by ethylnitrosourea. Proc. Natl. Acad. Sci. USA.

[54] Galvao R.P., Zong H. (2013). Inflammation and Gliomagenesis: Bi-Directional Communication at Early and Late Stages of Tumor Progression. Curr. Pathobiol. Rep..

[55] Blaylock R.L. (2013). Immunoexcitatory mechanisms in glioma proliferation, invasion and occasional metastasis. Surg. Neurol. Int..

[56] Conti A., Guli C., La Torre D., Tomasello C., Angileri F.F., Aguennouz M. (2010). Role of inflammation and oxidative stress mediators in gliomas. Cancers.

[57] Hodson L., Harnden K.E., Roberts R., Dennis A.L., Frayn K.N. (2010). Does the DASH diet lower blood pressure by altering peripheral vascular function?. J. Hum. Hypertens..

[58] Azadbakht L., Surkan P.J., Esmaillzadeh A., Willett W.C. (2011). The Dietary Approaches to Stop Hypertension eating plan affects C-reactive protein, coagulation abnormalities, and hepatic function tests among type 2 diabetic patients. J. Nutr..

[59] Marcason W. (2015). What Are the Components to the MIND Diet?. J. Acad. Nutr. Diet..

[60] Morris M.C., Tangney C.C., Wang Y., Sacks F.M., Barnes L.L., Bennett D.A., Aggarwal N.T. (2015). MIND diet slows cognitive decline with aging. Alzheimers Dement..

[61] Aghamohammadi V., Salari-Moghaddam A., Benisi-Kohansal S., Taghavi M., Azadbakht L., Esmaillzadeh A. (2021). Adherence to the MIND Diet and Risk of Breast Cancer: A Case-control Study. Clin. Breast Cancer.

[62] Sheikhhossein F., Imani H., Amini M.R., Hosseini F., Shab-Bidar S. (2021). The association between adherence to MIND diet and risk of breast cancer: A case-control study. Int. J. Clin. Pract..

[63] Soltani S., Shayanfar M., Benisi-Kohansal S., Mohammad-Shirazi M., Sharifi G., Djazayeri A., Esmaillzadeh A. (2022). Adherence to the MIND diet in relation to glioma: A case-control study. Nutr. Neurosci..

[64] Lehrer S. (2018). Glioma and Alzheimer’s Disease. J. Alzheimers Dis. Rep..

[65] de la O V., Zazpe I., Martinez J.A., Santiago S., Carlos S., Zulet M.A., Ruiz-Canela M. (2021). Scoping review of Paleolithic dietary patterns: A definition proposal. Nutr. Res. Rev..

[66] de la O V., Zazpe I., Goni L., Santiago S., Martin-Calvo N., Bes-Rastrollo M., Martinez J.A., Martinez-Gonzalez M.A., Ruiz-Canela M. (2022). A score appraising Paleolithic diet and the risk of cardiovascular disease in a Mediterranean prospective cohort. Eur. J. Nutr..

[67] Whalen K.A., Judd S., McCullough M.L., Flanders W.D., Hartman T.J., Bostick R.M. (2017). Paleolithic and Mediterranean Diet Pattern Scores Are Inversely Associated with All-Cause and Cause-Specific Mortality in Adults. J. Nutr..

[68] Klement R.J., Koebrunner P.S., Krage K., Weigel M.M., Sweeney R.A. (2020). Short-term effects of a Paleolithic lifestyle intervention in breast cancer patients undergoing radiotherapy: A pilot and feasibility study. Med. Oncol..

[69] Fenton T.R., Fenton C.J. (2016). Paleo diet still lacks evidence. Am. J. Clin. Nutr..

[70] Reuter S., Gupta S.C., Chaturvedi M.M., Aggarwal B.B. (2010). Oxidative stress, inflammation, and cancer: How are they linked?. Free Radic. Bio. Med..

[71] Nowsheen S., Aziz K., Kryston T.B., Ferguson N.F., Georgakilas A. (2012). The interplay between inflammation and oxidative stress in carcinogenesis. Curr. Mol. Med..

[72] Willett W., Rockstrom J., Loken B., Springmann M., Lang T., Vermeulen S., Garnett T., Tilman D., DeClerck F., Wood A. (2019). Food in the Anthropocene: The EAT-Lancet Commission on healthy diets from sustainable food systems. Lancet.

[73] Montejano V.R., Schulz C.A., van de Locht K., Oluwagbemigun K., Alexy U., Nothlings U. (2022). Associations of Adherence to a Dietary Index Based on the EAT-Lancet Reference Diet with Nutritional, Anthropometric, and Ecological Sustainability Parameters: Results from the German DONALD Cohort Study. J. Nutr..

[74] Cacau L.T., De Carli E., de Carvalho A.M., Lotufo P.A., Moreno L.A., Bensenor I.M., Marchioni D.M. (2021). Development and Validation of an Index Based on EAT-Lancet Recommendations: The Planetary Health Diet Index. Nutrients.

[75] Marchioni D.M., Cacau L.T., De Carli E., Carvalho A.M., Rulli M.C. (2022). Low Adherence to the EAT-Lancet Sustainable Reference Diet in the Brazilian Population: Findings from the National Dietary Survey 2017–2018. Nutrients.

[76] Chen Y., Chai L. (2022). How Far Are We from the Planetary Health Diet? A Threshold Regression Analysis of Global Diets. Foods.

[77] Malmir H., Shayanfar M., Mohammad-Shirazi M., Tabibi H., Sharifi G., Esmaillzadeh A. (2019). Patterns of nutrients intakes in relation to glioma: A case-control study. Clin. Nutr..

[78] Zhang D., Chen J., Wang J., Gong S., Jin H., Sheng P., Qi X., Lv L., Dong Y., Hou L. (2016). Body mass index and risk of brain tumors: A systematic review and dose-response meta-analysis. Eur. J. Clin. Nutr..

[79] Rieger J., Bahr O., Maurer G.D., Hattingen E., Franz K., Brucker D., Walenta S., Kammerer U., Coy J.F., Weller M. (2014). ERGO: A pilot study of ketogenic diet in recurrent glioblastoma. Int. J. Oncol..

[80] Porper K., Shpatz Y., Plotkin L., Pechthold R.G., Talianski A., Champ C.E., Furman O., Shimoni-Sebag A., Symon Z., Amit U. (2021). A Phase I clinical trial of dose-escalated metabolic therapy combined with concomitant radiation therapy in high-grade glioma. J. Neuro-Oncol..

[81] Zhao J., Li Z., Gao Q., Zhao H., Chen S., Huang L., Wang W., Wang T. (2021). A review of statistical methods for dietary pattern analysis. Nutr. J..

[82] Panagiotakos D.B., Pitsavos C., Stefanadis C. (2006). Dietary patterns: A Mediterranean diet score and its relation to clinical and biological markers of cardiovascular disease risk. Nutr. Metab. Cardiovas..

[83] Vogt T.M., Appel L.J., Obarzanek E., Moore T.J., Vollmer W.M., Svetkey L.P., Sacks F.M., Bray G.A., Cutler J.A., Windhauser M.M. (1999). Dietary Approaches to Stop Hypertension: Rationale, design, and methods. DASH Collaborative Research Group. J. Am. Diet. Assoc..

[84] Bertoia M.L., Triche E.W., Michaud D.S., Baylin A., Hogan J.W., Neuhouser M.L., Tinker L.F., Van Horn L., Waring M.E., Li W. (2014). Mediterranean and Dietary Approaches to Stop Hypertension dietary patterns and risk of sudden cardiac death in postmenopausal women. Am. J. Clin. Nutr..

