# Supplementary material for: Adherence to Healthy Dietary Patterns and Glioma: A Matched Case-Control Study
Source: Nutrients. 2023 Nov 23;15(23):4886. doi: 10.3390/nu15234886 (PMC10708472; doi:10.3390/nu15234886)
Supplement: Supplementary file 1 [file nutrients-15-04886-s001.zip › nutrients-2700173-supplementary.pdf]

Supplementary material A

Supplemental methods: Components and scoring of the dietary pattern scores

Mediterranean diet

According to previous reports, the Mediterranean diet score involved a total of 11 food groups. Intakes for the following food groups should be used to calculate diet scores: grains, fruits, vegetables, legumes, potatoes, fish, red and processed meats, poultry, dairy products, olive oil, and alcohol. These foods were then scored according to the recommended intake, with each food group scoring in the range of 0-5 points. The intake of grains, fruits, vegetables, legumes, potatoes, fish, and olive oil was positively correlated with the scores, while the scores of red and processed meat, poultry and dairy products were distributed in opposite proportions of intake. In particular, 5 points were given for those who drank less than 36g of alcohol per day, 0 points for those who drank more than 84g of alcohol per day or did not consume alcohol, and 4-1 points for those who drank 36g to 84g of alcohol per day. Therefore, the total score ranged between 0-55 points [1].

DASH diet

The DASH diet scores developed based on the DASH study involved a total of 8 food groups [2]. Intake of fruits, vegetables, nuts and legumes, dairy products, red and processed meats, sugary drinks, whole grains, and sodium was divided into quintiles after adjusting for energy. The scores from the lowest to the highest quintiles in the following food groups are scored 1-5 points: fruits, vegetables, nuts and legumes, dairy

products, and whole grains. Intake of the rest of the foods (sodium, red and processed meat, and sugary drinks) was inverted with the score (minimum to maximum intake quintile was 5-1 points). Therefore, the total score ranges from 8-40 points [3].

MIND diet

Based on the Mediterranean diet and the DASH diet [4], the diet pattern summarized 15 groups of foods closely related to the brain and nerves, including 10 healthy food groups of green leafy vegetables, other vegetables, nuts, berries, legumes, whole grains, fish, poultry, olive oil and wine, and 5 groups of unhealthy foods of red and processed meats, butter, cheese, pastries and sweets, and fried foods. If the study subjects identified olive oil as a daily cooking oil, the score was 1, otherwise, the score was 0. The remaining 14 foods were scored based on recommended intakes, with each scoring 0, 0.5, or 1. Since no food cooking methods were collected in this study, fried foods were not included in the evaluation scope of this study. Therefore, the total score ranged from 0-14 points.

Paleolithic diet

The Paleolithic diet was a dietary pattern based on unprocessed animal and plant food, which was formed by referring to the human lifestyle in the Paleolithic Age [5]. This diet pattern involves a total of 11 food groups in the paleo diet. Fruits, nuts, vegetables, eggs, meat, and fish were advocated, while grains, dairy products, legumes, cooking ingredients (added sugars, salt, and refined fats), and processed foods were considered to be discouraged. Divided into quintiles according to the intake of each food group, the group of foods that advocated intake was scored 1-5 points sequentially according

to the lowest to highest intake, and the group of foods that did not encourage intake scored the opposite. Each food group had the same weight, so its total score ranged from 11-55 points.

The PH diet

This dietary pattern was developed in response to dietary recommendations made by the EAT-Lancet Commission [6]. The PH diet divided food groups into four areas: adequacy, optimum, ratio, and moderation. Adequacy components were foods that did not have a serious health impact even when consumed too much, including nuts and peanuts, legumes, fruits, total vegetables, and whole grains. Optimum components were that food intake should be moderate, and too much or too little could affect health, including eggs, fish and seafood, tubers and potatoes, dairy products, and vegetable oils. In contrast to adequacy components, moderation components were foods that were healthier with a smaller intake, including red meat, chicken and substitutes, animal fats, and added sugars. The ratio components mainly included the ratio of dark green vegetables and red and orange vegetables relative to the total intake of vegetables. Unlike other dietary patterns, this diet score was evaluated based on the ratio of energy provided by different food groups to the total energy. According to the recommended range of caloric density for each food group, the corresponding score was obtained, and each food group scored 0-10 points (0-5 points for each item in the ratio component), so its total score ranged is 0-150 points [7].

References:

1. Panagiotakos, D.B.; Pitsavos, C.; Stefanadis, C. Dietary patterns: a Mediterranean diet score and its relation to clinical and biological markers of cardiovascular disease risk. Nutr Metab Cardiovas 2006, 16, 559-568, doi:10.1016/j.numecd.2005.08.006.
2. Vogt, T.M.; Appel, L.J.; Obarzanek, E.; Moore, T.J.; Vollmer, W.M.; Svetkey, L.P.; Sacks, F.M.; Bray, G.A.; Cutler, J.A.; Windhauser, M.M., et al. Dietary Approaches to Stop Hypertension: rationale, design, and methods. DASH Collaborative Research Group. J Am Diet Assoc 1999, 99, S12-S18, doi:10.1016/s0002-8223(99)00411-3.
3. Bertoia, M.L.; Triche, E.W.; Michaud, D.S.; Baylin, A.; Hogan, J.W.; Neuhouser, M.L.; Tinker, L.F.; Van Horn, L.; Waring, M.E.; Li, W., et al. Mediterranean and Dietary Approaches to Stop Hypertension dietary patterns and risk of sudden cardiac death in postmenopausal women. Am J Clin Nutr 2014, 99, 344-351, doi:10.3945/ajcn.112.056135.
4. Morris, M.C.; Tangney, C.C.; Wang, Y.; Sacks, F.M.; Barnes, L.L.; Bennett, D.A.; Aggarwal, N.T. MIND diet slows cognitive decline with aging. Alzheimers Dement 2015, 11, 1015-1022, doi:10.1016/j.jalz.2015.04.011.
5. de la O, V.; Zazpe, I.; Goni, L.; Santiago, S.; Martin-Calvo, N.; Bes-Rastrollo, M.; Martinez, J.A.; Martinez-Gonzalez, M.A.; Ruiz-Canela, M. A score appraising Paleolithic diet and the risk of cardiovascular disease in a Mediterranean prospective cohort. Eur J Nutr 2022, 61, 957-971, doi:10.1007/s00394-021-02696-9.

6. Willett, W.; Rockstrom, J.; Loken, B.; Springmann, M.; Lang, T.; Vermeulen, S.; Garnett, T.; Tilman, D.; DeClerck, F.; Wood, A., et al. Food in the Anthropocene: the EAT-Lancet Commission on healthy diets from sustainable food systems. Lancet 2019, 393, 447-492, doi:10.1016/S0140-6736(18)31788-4.
7. Cacau, L.T.; De Carli, E.; de Carvalho, A.M.; Lotufo, P.A.; Moreno, L.A.; Bensenor, I.M.; Marchioni, D.M. Development and Validation of an Index Based on EAT-Lancet Recommendations: The Planetary Health Diet Index. Nutrients 2021, 13, doi:10.3390/nu13051698.

Supplementary material B

Table S1. Dietary items in the food frequency questionnaire

Table S2. Basic characteristics of all study participants

Table S3. Adjusted ORs and 95% CIs for the association between dietary pattern scores and glioma of different grades

Table S4. Factor loading matrix for the major dietary patterns derived from PCA

Table S5. Adjusted ORs and 95% CIs for gliomas in subgroups

Table S6. Dietary patterns influenced the proportion of gliomas mediated by BMI

Figure S1. The flow chart of the case-control population matching

Table S1. Dietary items in the food frequency questionnaire

Food groups(n)	Items
Refined grains (9)	Rice, steamed buns, scones, porridge, deep-fried dough sticks, noodles, instant noodles, vermicelli, dumplings
Whole grains (2)	Corn, Millet and oats, etc
Tubers (2)	Potatoes, sweet potatoes
Legume and Products (5)	Soy, soy milk, tofu, other soy products, other legumes
Vegetables (23)	Green leafy vegetables, kale, cabbage, celery, eggplant, cucumber, white gourd, pumpkin, carrot, radish, green shoot, broccoli, bell pepper, pepper, tomato, lotus root, chive, garlic moss, bitter melon, onion, green bean, zucchini, cauliflower
Fungi and algae (4)	Mushrooms, Auricularia auricula, tremella, kelp
Fruits (20)	Apple, pear, orange, banana, peach, watermelon, melon, grape, pineapple, kiwi fruit, strawberries, cherries, blueberries, mulberries, shaddock, hawthorn, pitaya, date, mango, cherry tomato
Red meat (4)	pork (muscle), pork (fat & muscle), beef, mutton
Poultry (2)	Chicken, duck
Animal viscera (4)	liver, blood, intestine, other viscera
Fish and seafood (5)	Freshwater fish, sea fish, shrimp, crab, mollusk
Egg (1)	Fresh egg
Dairy products (4)	Milk, yogurt, milk powder, cheese
Nut (4)	Peanuts, sunflower seeds, walnuts, other nuts
Sweet food (5)	Bread, honey, biscuit, cake, Chinese pastry
Sugary drink (3)	Carbonated drinks, fruit and vegetable drinks, functional drinks
Tea and coffee (3)	Green tea, black tea, coffee
Salt (1)	Salt
Oils (3)	Animal oil olive oil, other vegetable oil
Curing and processed products (6)	Sauerkraut, pickle, fermented bean curd, bacon, sausage and other smoked meat, salted duck eggs, preserved eggs
Alcohol (4)	Beer, spirit, wine, rice wine

Table S2. Basic characteristics of all study participants

	**Case(n=506)**	**Control(n=506)**	***p*** [a]
Age(years)	42.62±13.09	41.15±12.85	0.072
Sex, n(%)			1.000
Male	284(56.1)	284(56.1)	
Female	222(43.9)	222(43.9)	
BMI(kg/m^2)	24.03±3.25	23.05±3.27	<0.001
High-risk residential area, n(%)			0.534
Yes	108(21.3)	100(19.8)	
No	398(78.7)	406(80.2)	
Occupation, n(%)			0.024
Manual workers	134(26.5)	103(20.4)	
Mental workers	265(52.4)	306(60.5)	
Others	107(21.1)	97(19.1)	
Education level, n(%)			<0.001
Primary school and below	35(6.9)	13(2.6)	
Middle school	210(41.5)	127(25.1)	
University and above	261(51.6)	366(72.3)	
Household income, n(%)			<0.001
<3,000 ¥/month	49(9.7)	92(18.2)	
3000-10,000 ¥/month	384(75.9)	249(49.2)	
>10,000 ¥/month	73(14.4)	165(32.6)	
Smoking status, n(%)			0.039
Never smoking	354(70.0)	381(75.3)	
Former smoking	65(12.8)	41(8.1)	
Current smoking	87(17.2)	84(16.6)	
Alcohol consumption, n(%)			<0.001
Never drinkers	324(64.1)	285(56.4)	
Occasional drinkers	65(12.8)	153(30.2)	
Frequent drinkers	117(23.1)	68(13.4)	
History of allergies, n(%)			<0.001
Yes	39(7.7)	74(14.6)	
No	467(92.3)	432(85.4)	
History of head trauma, n(%)			0.474
Yes	57(11.3)	50(9.9)	
No	449(88.7)	456(90.1)	
Family history of cancer, n(%)			0.001
Yes	152(30.0)	107(21.1)	
No	354(70.0)	399(78.9)	
Physical Activity, n(%)			<0.001
Low	69(13.6)	232(45.8)	
Moderate	209(41.3)	184(36.4)	
Violent	228(45.1)	90(17.8)	

[a] *p*-values were derived from Student's t-tests for continuous variables according to the data distribution and the chi-square test for the classified variables.

Table S3. Adjusted ORs and 95% CIs for the association between dietary pattern scores and glioma of different grades

Glioma grading [a]	Model 1[b]	*p*	Model 2[c]	*p*
**Low grade**				
Mediterranean Diet	0.94(0.88-0.99)	0.037	0.85(0.75-0.97)	0.013
DASH Diet	0.82(0.75-0.89)	<0.001	0.63(0.47-0.85)	0.002
MIND Diet	0.68(0.53-0.87)	0.002	0.60(0.36-0.99)	0.047
Paleolithic Diet	0.82(0.75-0.90)	<0.001	0.72(0.59-0.88)	0.002
PH Diet	0.99(0.97-1.02)	0.692	0.98(0.94-1.03)	0.445
**High grade**				
Mediterranean Diet	0.94(0.91-0.97)	<0.001	0.90(0.84-0.96)	0.002
DASH Diet	0.85(0.82-0.89)	<0.001	0.78(0.70-0.86)	<0.001
MIND Diet	0.66(0.57-0.76)	<0.001	0.48(0.34-0.69)	<0.001
Paleolithic Diet	0.87(0.84-0.90)	<0.001	0.80(0.73-0.88)	<0.001
PH Diet	0.99(0.98-1.00)	0.164	0.99(0.96-1.01)	0.352

Note: Grade I and III gliomas were small in sample size and were not analyzed separately.

[a.] The results came from dietary scores as continuous variables.

[b.] Model 1: Unadjusted model.

[C.] Model 2: Adjusted for age, BMI, occupation, education level, household income, high-risk residential areas, smoking status, alcohol consumption, history of allergies, history of head trauma, family history of cancer, physical activity, and energy intake.

Table S4. Factor loading matrix for the major dietary patterns derived from PCA

	Factor 1	Factor 2	Factor 3	Factor 4	Factor 5	Factor 6
Refined grains		0.520				0.365
Whole grains	0.527					
Legume and Products	0.553					
Tubers	0.451		0.414			
Vegetables	0.759					
Fungi and algae	0.682					
Fruits	0.646					
Red meat		0.461				
Poultry			-0.508			
Animal viscera				0.436		
Fish and seafood	0.544					
Egg					0.434	
Dairy products	0.412		-0.377			
Nut	0.429					
Sweet food	0.378					
Sugary drink					-0.446	
Tea and coffee				0.390	0.471	
Salt		0.687				
Oils		0.749				
Curing and processed products					-0.401	
Alcohol		0.363		0.566		0.415

Table S5. Adjusted ORs and 95% CIs for gliomas in subgroups

Subgroup[a]	Model 1[d]	*p*	Model 2[e]	*p*
**Age**				
**≤40(n=500)**				
Mediterranean Diet	0.96(0.93-0.99)	0.026	1.00(0.96-1.05)	0.980
DASH Diet	0.89(0.86-0.92)	<0.001	0.89(0.85-0.94)	<0.001
MIND Diet	0.74(0.64-0.86)	<0.001	0.65(0.53-0.80)	<0.001
Paleolithic Diet	0.87(0.84-0.91)	<0.001	0.85(0.81-0.90)	<0.001
PH Diet	1.01(0.99-1.02)	0.313	1.01(0.99-1.03)	0.216
**>40(n=512)**				
Mediterranean Diet	0.90(0.86-0.93)	<0.001	0.90(0.86-0.94)	<0.001
DASH Diet	0.81(0.78-0.85)	<0.001	0.80(0.76-0.85)	<0.001
MIND Diet	0.53(0.45-0.63)	<0.001	0.48(0.39-0.60)	<0.001
Paleolithic Diet	0.85(0.81-0.89)	<0.001	0.85(0.81-0.90)	<0.001
PH Diet	0.97(0.95-0.98)	<0.001	0.97(0.95-0.99)	0.001
**Sex**				
**Male(n=568)**				
Mediterranean Diet	0.95(0.92-0.98)	0.001	0.96(0.92-0.99)	0.019
DASH Diet	0.84(0.81-0.87)	<0.001	0.83(0.79-0.87)	<0.001
MIND Diet	0.69(0.60-0.79)	<0.001	0.59(0.49-0.71)	<0.001
Paleolithic Diet	0.87(0.84-0.91)	<0.001	0.87(0.83-0.91)	<0.001
PH Diet	0.99(0.97-1.00)	0.041	0.98(0.97-1.00)	0.045
**Female(n=444)**				
Mediterranean Diet	0.92(0.89-0.96)	<0.001	0.91(0.86-0.97)	0.002
DASH Diet	0.85(0.82-0.89)	<0.001	0.86(0.82-0.92)	<0.001
MIND Diet	0.58(0.49-0.68)	<0.001	0.54(0.43-0.69)	<0.001
Paleolithic Diet	0.83(0.79-0.87)	<0.001	0.83(0.77-0.88)	<0.001
PH Diet	0.99(0.98-1.01)	0.467	1.00(0.98-1.02)	0.994
**BMI**				
**≤23.31(n=506)**				
Mediterranean Diet	0.97(0.94-1.01)	0.090	0.97(0.93-1.02)	0.266
DASH Diet	0.87(0.83-0.90)	<0.001	0.87(0.83-0.92)	<0.001
MIND Diet	0.68(0.59-0.79)	<0.001	0.61(0.50-0.75)	<0.001
Paleolithic Diet	0.88(0.84-0.91)	<0.001	0.87(0.83-0.92)	<0.001
PH Diet	1.00(0.99-1.01)	0.977	1.00(0.99-1.02)	0.658
**>23.31(n=506)**				
Mediterranean Diet	0.91(0.88-0.94)	<0.001	0.93(0.89-0.97)	0.002
DASH Diet	0.85(0.82-0.88)	<0.001	0.85(0.81-0.89)	<0.001
MIND Diet	0.59(0.50-0.70)	<0.001	0.55(0.45-0.68)	<0.001
Paleolithic Diet	0.85(0.81-0.89)	<0.001	0.83(0.79-0.88)	<0.001
PH Diet	0.98(0.96-0.99)	0.002	0.98(0.97-1.00)	0.083
**Occupation**				
**Manual workers(n=237)**				
Mediterranean Diet	0.95(0.90-0.99)	0.040	0.97(0.90-1.04)	0.340

DASH Diet	0.84(0.79-0.89)	<0.001	0.86(0.79-0.94)	0.001
MIND Diet	0.55(0.43-0.71)	<0.001	0.47(0.32-0.68)	<0.001
Paleolithic Diet	0.88(0.83-0.94)	<0.001	0.84(0.77-0.92)	<0.001
PH Diet	0.97(0.95-0.99)	0.004	0.98(0.94-1.01)	0.110
**Mental workers(n=571)**				
Mediterranean Diet	0.94(0.92-0.98)	<0.001	0.96(0.92-0.99)	0.039
DASH Diet	0.87(0.84-0.90)	<0.001	0.86(0.82-0.90)	<0.001
MIND Diet	0.71(0.62-0.81)	<0.001	0.63(0.53-0.75)	<0.001
Paleolithic Diet	0.88(0.84-0.91)	<0.001	0.87(0.83-0.91)	<0.001
PH Diet	1.00(0.99-1.01)	0.786	1.00(0.98-1.02)	0.899
**Others(n=204)**				
Mediterranean Diet	0.88(0.84-0.94)	<0.001	0.85(0.77-0.92)	<0.001
DASH Diet	0.83(0.78-0.88)	<0.001	0.80(0.72-0.88)	<0.001
MIND Diet	0.57(0.44-0.73)	<0.001	0.46(0.30-0.70)	<0.001
Paleolithic Diet	0.79(0.73-0.85)	<0.001	0.78(0.70-0.88)	<0.001
PH Diet	0.98(0.96-1.00)	0.118	0.97(0.94-1.01)	0.110
**Education level**				
**Middle school and below(n=385)** [b]				
Mediterranean Diet	0.92(0.88-0.96)	<0.001	0.94(0.89-0.99)	0.024
DASH Diet	0.84(0.80-0.88)	<0.001	0.86(0.81-0.92)	<0.001
MIND Diet	0.54(0.44-0.66)	<0.001	0.51(0.39-0.68)	<0.001
Paleolithic Diet	0.85(0.80-0.89)	<0.001	0.84(0.78-0.90)	<0.001
PH Diet	0.98(0.96-0.99)	0.011	0.99(0.97-1.01)	0.411
**University and above(n=627)**				
Mediterranean Diet	0.93(0.90-0.96)	<0.001	0.95(0.91-0.99)	0.011
DASH Diet	0.86(0.83-0.89)	<0.001	0.86(0.82-0.90)	<0.001
MIND Diet	0.69(0.60-0.79)	<0.001	0.63(0.53-0.74)	<0.001
Paleolithic Diet	0.87(0.84-0.91)	<0.001	0.87(0.83-0.91)	<0.001
PH Diet	0.99(0.98-1.01)	0.330	0.99(0.98-1.01)	0.388
**Household income**				
**<3,000 ¥/month(n=141)**				
Mediterranean Diet	1.02(0.95-1.08)	0.644	1.01(0.92-1.10)	0.838
DASH Diet	0.89(0.82-0.96)	0.002	0.89(0.79-0.99)	0.028
MIND Diet	0.75(0.55-1.01)	0.059	0.64(0.41-0.99)	0.043
Paleolithic Diet	0.88(0.81-0.96)	0.003	0.86(0.76-0.96)	0.009
PH Diet	1.00(0.98-1.03)	0.840	1.03(0.99-1.07)	0.120
**3,000-10,000 ¥/month(n=633)**				
Mediterranean Diet	0.90(0.87-0.93)	<0.001	0.91(0.88-0.95)	<0.001
DASH Diet	0.84(0.81-0.88)	<0.001	0.83(0.79-0.87)	<0.001
MIND Diet	0.57(0.49-0.67)	<0.001	0.49(0.41-0.60)	<0.001
Paleolithic Diet	0.87(0.84-0.90)	<0.001	0.86(0.82-0.90)	<0.001
PH Diet	0.99(0.98-1.00)	0.070	0.99(0.98-1.01)	0.446
**>10,000 ¥/month(n=238)**				
Mediterranean Diet	0.99(0.94-1.04)	0.695	0.99(0.93-1.07)	0.971

DASH Diet	0.90(0.85-0.95)	<0.001	0.92(0.86-0.99)	0.019
MIND Diet	0.75(0.61-0.92)	0.006	0.80(0.61-1.04)	0.100
Paleolithic Diet	0.85(0.79-0.91)	<0.001	0.83(0.76-0.91)	<0.001
PH Diet	0.98(0.96-1.00)	0.113	0.98(0.96-1.01)	0.147
**Smoking status**				
**Never smoking(n=735)**				
Mediterranean Diet	0.94(0.91-0.97)	<0.001	0.95(0.92-0.99)	0.010
DASH Diet	0.87(0.84-0.90)	<0.001	0.88(0.85-0.91)	<0.001
MIND Diet	0.68(0.60-0.76)	<0.001	0.63(0.54-0.74)	<0.001
Paleolithic Diet	0.86(0.83-0.89)	<0.001	0.85(0.81-0.89)	<0.001
PH Diet	1.00(0.99-1.01)	0.650	1.00(0.98-1.01)	0.898
**Smoking(n=277)** [c]				
Mediterranean Diet	0.93(0.89-0.97)	0.002	0.93(0.87-0.98)	0.011
DASH Diet	0.80(0.75-0.86)	<0.001	0.76(0.70-0.83)	<0.001
MIND Diet	0.55(0.44-0.70)	<0.001	0.38(0.27-0.54)	<0.001
Paleolithic Diet	0.88(0.83-0.93)	<0.001	0.87(0.81-0.93)	<0.001
PH Diet	0.97(0.96-0.99)	0.005	0.97(0.95-0.99)	0.018
**History of allergies**				
**Yes(n=113)**				
Mediterranean Diet	0.91(0.85-0.99)	0.019	0.94(0.85-1.04)	0.244
DASH Diet	0.88(0.81-0.95)	0.001	0.86(0.77-0.95)	0.004
MIND Diet	0.67(0.48-0.92)	0.015	0.52(0.33-0.83)	0.006
Paleolithic Diet	0.93(0.85-1.02)	0.138	0.92(0.83-1.03)	0.144
PH Diet	0.98(0.95-1.01)	0.187	0.98(0.94-1.02)	0.222
**No(n=899)**				
Mediterranean Diet	0.94(0.92-0.97)	<0.001	0.95(0.92-0.98)	0.001
DASH Diet	0.85(0.83-0.88)	<0.001	0.86(0.82-0.89)	<0.001
MIND Diet	0.64(0.57-0.72)	<0.001	0.58(0.50-0.68)	<0.001
Paleolithic Diet	0.86(0.83-0.89)	<0.001	0.85(0.82-0.88)	<0.001
PH Diet	0.99(0.98-1.00)	0.087	1.00(0.98-1.01)	0.469
**Family history of cancer**				
**Yes(n=259)**				
Mediterranean Diet	0.93(0.89-0.98)	0.005	0.94(0.88-1.01)	0.091
DASH Diet	0.86(0.81-0.91)	<0.001	0.83(0.77-0.90)	<0.001
MIND Diet	0.74(0.59-0.91)	0.006	0.70(0.52-0.94)	0.019
Paleolithic Diet	0.85(0.80-0.91)	<0.001	0.81(0.74-0.89)	<0.001
PH Diet	1.00(0.98-1.02)	0.706	1.00(0.97-1.02)	0.751
**No(n=753)**				
Mediterranean Diet	0.94(0.91-0.97)	<0.001	0.95(0.92-0.99)	0.005
DASH Diet	0.85(0.83-0.88)	<0.001	0.86(0.83-0.89)	<0.001
MIND Diet	0.61(0.54-0.69)	<0.001	0.53(0.45-0.62)	<0.001
Paleolithic Diet	0.86(0.84-0.89)	<0.001	0.86(0.83-0.90)	<0.001
PH Diet	0.99(0.98-1.00)	0.010	0.99(0.98-1.01)	0.196
**Physical Activity**				

**Low(n=301)**				
Mediterranean Diet	0.96(0.91-1.01)	0.085	0.95(0.89-1.01)	0.120
DASH Diet	0.91(0.86-0.96)	0.001	0.88(0.82-0.95)	0.001
MIND Diet	0.97(0.79-1.19)	0.743	0.94(0.71-1.26)	0.696
Paleolithic Diet	0.95(0.89-1.01)	0.099	0.93(0.86-1.00)	0.060
PH Diet	1.02(0.99-1.04)	0.156	1.02(0.99-1.05)	0.122
**Moderate(n=393)**				
Mediterranean Diet	0.93(0.90-0.97)	<0.001	0.94(0.90-0.99)	0.014
DASH Diet	0.86(0.82-0.90)	<0.001	0.86(0.81-0.91)	<0.001
MIND Diet	0.65(0.55-0.77)	<0.001	0.58(0.47-0.72)	<0.001
Paleolithic Diet	0.87(0.83-0.91)	<0.001	0.86(0.81-0.91)	<0.001
PH Diet	0.98(0.97-0.99)	0.021	0.98(0.96-1.00)	0.085
**Violent(n=318)**				
Mediterranean Diet	0.93(0.89-0.98)	0.004	0.94(0.88-0.99)	0.040
DASH Diet	0.81(0.76-0.86)	<0.001	0.82(0.77-0.89)	<0.001
MIND Diet	0.37(0.28-0.48)	<0.001	0.34(0.24-0.48)	<0.001
Paleolithic Diet	0.82(0.77-0.87)	<0.001	0.81(0.75-0.87)	<0.001
PH Diet	0.99(0.97-1.01)	0.226	0.98(0.96-1.01)	0.169

[a]. Unconditional logistic regression model was used for subgroup analysis. The results came from dietary scores as continuous variables.

[b]. Middle school and below included primary school and below and middle school.

[c]. Smoking included former smoking, and current smoking.

[d]. Model 1: Unadjusted model.

[e]. Adjusted covariates in model 2 (except for corresponding hierarchical variables).

Table S6. Dietary patterns influenced the proportion of gliomas mediated by BMI

Diet pattern[a]	Total effect	ADE	ACME	Proportion mediated (%)
Mediterranean Diet	-0.07(-0.10, -0.04)	-0.07(-0.10, -0.04)	-0.002(-0.006, 0)	2.50
p	<0.001	<0.001	0.340	0.340
DASH Diet	-0.14(-0.15, -0.11)	-0.13(-0.15, -0.11)	-0.004(-0.01, 0)	2.82
p	<0.001	<0.001	0.014	0.014
MIND Diet	-0.11(-0.13, -0.09)	-0.11(-0.13, -0.08)	-0.004(-0.01, 0)	3.24
p	<0.001	<0.001	0.008	0.008
Paleolithic Diet	-0.12(-0.14, -0.10)	-0.12(-0.14, -0.10)	-0.003(-0.01, 0)	2.56
p	<0.001	<0.001	0.056	0.056

Note: ACME: average causal mediation effects; ADE: average direct effects

[a.] Adjusted for age, occupation, education level, household income, high-risk residential areas, smoking status, alcohol consumption, history of allergies, history of head trauma, family history of cancer, physical activity, and energy intake.

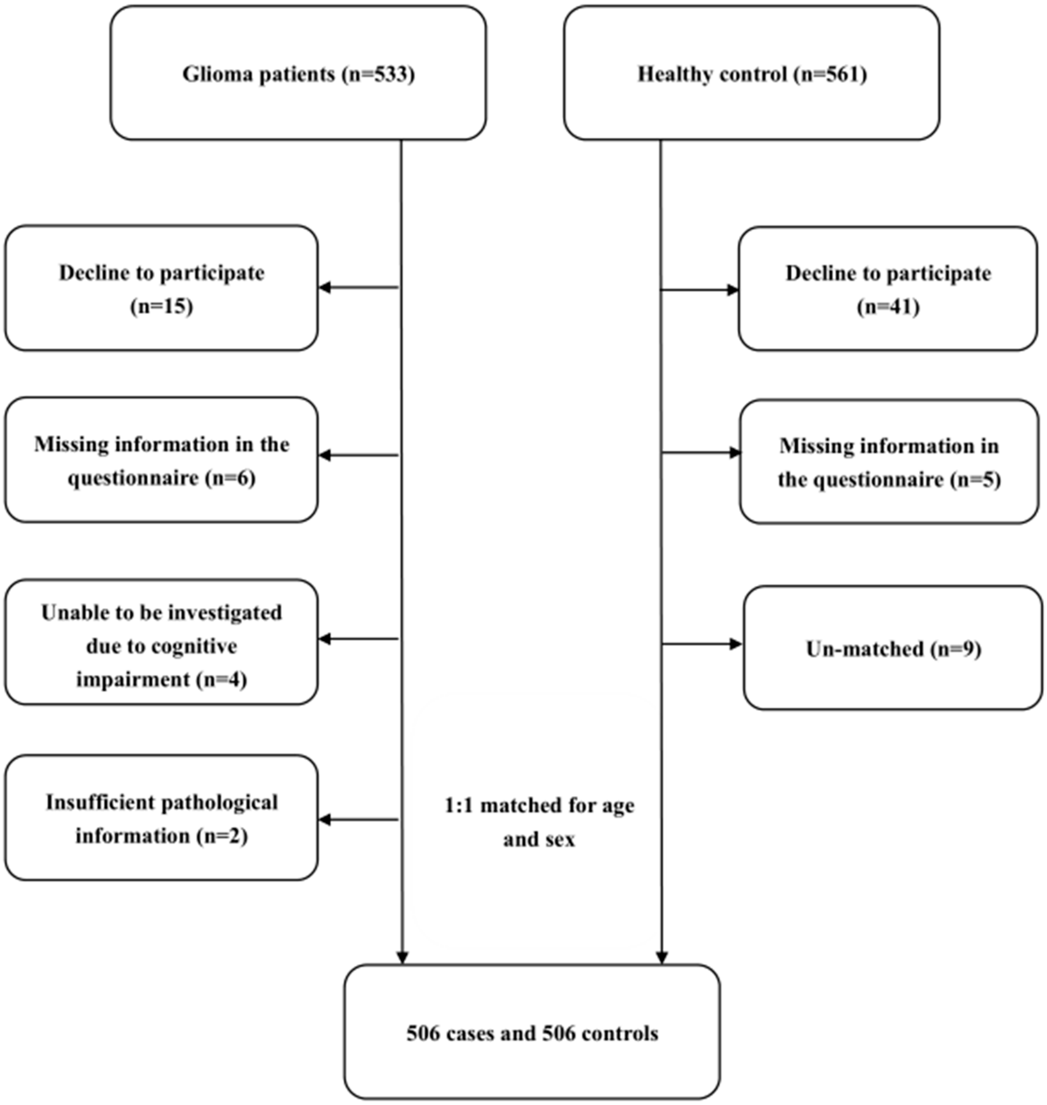

Figure S1. The flow chart of the case-control population matching
